# Frosting on porous membranes in energy exchangers

**DOI:** 10.1098/rsta.2024.0365

**Published:** 2025-07-17

**Authors:** Melanie Fauchoux, Amirreza Mahmoudi, Siddhartha Gollamudi, Shirin Niroomand, Pooya Navid, Albin Joseph, Carey Simonson

**Affiliations:** ^1^ Department of Mechanical Engineering, University of Saskatchewan, Saskatoon, Saskatchewan, Canada

**Keywords:** frosting, porous membrane, moisture transfer, energy exchangers, LAMEE

## Abstract

A liquid-to-air membrane energy exchanger (LAMEE) is a device that uses a semi-permeable membrane to transfer heat and moisture between an air stream and a liquid stream. In cold climates, they can be used to dehumidify an air stream and reduce or even prevent frost formation inside the exchanger. Understanding the mechanisms of frosting and the frost limits on membranes is essential for advancing the applications of LAMEEs in cold conditions. This paper combines a review of previously published research on the growth of frost on a membrane surface, compared to an impermeable surface, along with new experimental results that extend the applications of the frost research to cover more air temperature and relative humidity (RH) conditions. Frost limit maps are created for various operating conditions using an analytical model and verified with experimental results. These maps indicate the liquid temperature and air RH values that will result in frost conditions on the membrane surface. It was found that when the air temperature is 23°C, the liquid temperature could be lowered by 2°C–3°C at a constant RH level without frost appearing on the surface as compared to an impermeable surface, and when the air temperature is 0°C, the liquid temperature could be lowered by approximately 5°C compared to the impermeable surface. The results show that a porous membrane has great potential to create frost-free energy exchangers.

This article is part of the theme issue ‘Heat and mass transfer in frost and ice’.

## 1. Introduction

A liquid-to-air membrane energy exchanger (LAMEE) is an energy recovery device that can transfer both heat and moisture between an air stream and a liquid stream. The two streams are separated by a membrane, which allows for the simultaneous transfer of both sensible heat and moisture between the two streams. One application of a LAMEE is to dehumidify or humidify an airstream. Conventional methods for dehumidifying an airstream require that the air be cooled below its dewpoint temperature, causing the moisture in the air to condense on the surface, and then the air is reheated to the desired temperature. A LAMEE can dehumidify the air directly, without having to cool and reheat. Also, the condensation must be removed from the conventional device so as not to cause problems in the device, particularly in conditions where the surface temperature is cold, and the condensation may turn into frost or ice. The formation of frost or ice in an energy exchanger can cause significant damage to the surface of the exchanger and requires frequent defrost cycles, which reduces the effectiveness of the exchanger and can lead to increased energy consumption and emissions.

A LAMEE can be used to humidify an airstream using water in the liquid channel, as water has an equilibrium relative humidity (RH) equal to 100% RH. In this case, the driving potential for moisture transfer will be from the liquid side to the air side. However, a LAMEE has more benefits when a liquid desiccant (LD), such as an aqueous salt solution, is used in the liquid channel. LDs have equilibrium RHs lower than 100% RH, and if a value lower than the air humidity level is used, moisture transfer will be from the airstream to the liquid channel. For example, at 25°C a saturated solution of MgCl_2_ has an equilibrium RH of 33% RH, and LiCl has an equilibrium RH of 11% RH. By controlling the concentration of the LD solution, the driving potential for moisture transfer can be increased, allowing for more moisture transfer and greater potential to dehumidify the air.

At the heart of a LAMEE is a semi-permeable membrane, which allows water vapour to transfer through, but is impermeable to liquids . These membranes may be categorized as dense or porous, depending on their pore size, and as hydrophobic or hydrophilic if they have a weak or strong affinity to water, respectively [1]. Porous membranes, which are the focus of this paper, have pore sizes on the order of 1 μm, while dense membranes have pore sizes on the order of 1 nm. The membrane used is hydrophobic, meaning that the water molecules have a stronger attraction to each other than the surface of the membrane, which prevents the LD from entering the pores of the membrane, only allowing water vapour to transfer through.

As mentioned, one of the main benefits of a LAMEE is its ability to dehumidify an airstream. This can be helpful in warm, humid climates, where the outdoor air is too humid to supply to a building, as high humidity levels in buildings can lead to damage of the building materials and cause occupant discomfort. It can also be beneficial in cold climates, where there is a risk of frost forming on the exchanger surface. As the water vapour transfers from the airstream, through the membrane into the liquid channel, the risk of frost formation on the surface of the membrane is reduced. One potential application of a LAMEE in cold climates is the coupling of a LAMEE with an air-source heat pump to prevent frost build up on the surface of the heat pump evaporator that is in contact with the cold outdoor air.

In this paper, frost growth on a porous membrane is detailed, along with the effect of moisture transfer through the membrane on the air conditions that lead to the onset of frost. Both experimental and analytical methods are used. The paper consists of a review of previously published work with the addition of new data, expanding on the test conditions used, and providing a new analysis of the results. All work presented was conducted by the authors at the University of Saskatchewan. The goal of this work is to understand the mechanisms of frost growth on porous membranes and the effects of moisture transfer through the membrane at the onset of frost growth.

## 2. Methods

### (a) Experimental methods

Understanding how frost grows on a porous membrane is critical to developing membrane-based energy exchangers that are frost-resistant. To that end, experiments have been conducted [2–4], and both an analytical and numerical model have been developed [5,6] to study frost growth on porous membranes and the reduction in frost formation attributed to moisture transfer through the membrane. A summary of these results, which were conducted using room temperature air (23°C) and varying LD solution temperatures, is presented, along with analytical results. New tests have been conducted using colder air (0°C), with high RH (50%–85% RH), and even colder LD temperatures to expand the application of the membrane exchangers to even colder climates.

**Figure 1.**
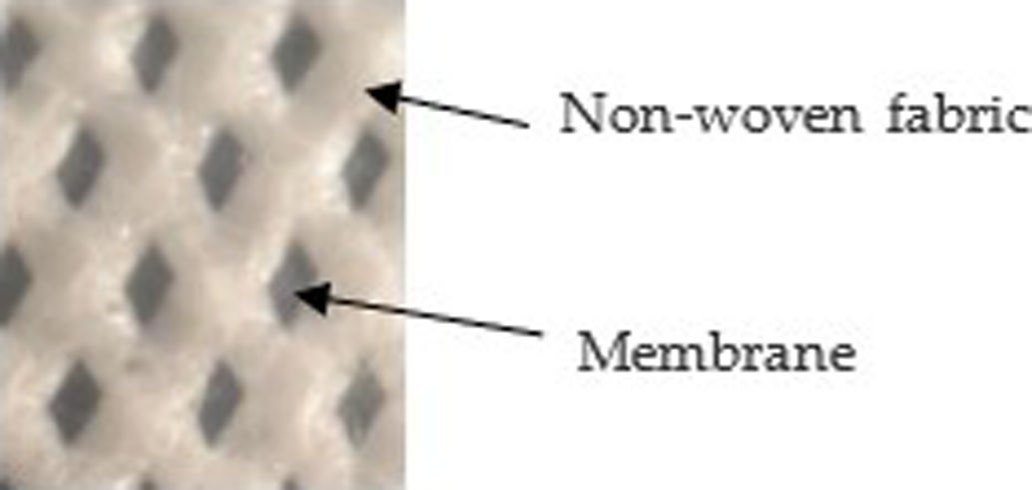
Photograph of the top view of the Propore^TM^ membrane.

The hydrophobic, porous membrane used in this study is Propore^TM^, which consists of a porous, permeable polypropylene membrane affixed to a non-woven polypropylene fabric, which provides support to the membrane. The membrane must be carefully stretched and glued to the test section to ensure no rips or tears occur, which may cause leakage during the testing. Figure 1 shows a close-up photograph of the membrane with the membrane and support fabric noted. The air side of the membrane has an uneven surface, which may affect the growth of frost. Propore^TM^ is commonly used in light-duty rainwear, medical packaging and in hospitals for disposable mattress and pillowcase covers.

The properties of the membrane that are relevant to this work are listed in table 1, including the membrane thickness (*L*), thermal conductivity (*k*_mem_), resistance to moisture transfer (*R_m_*_,mem_) and the direct contact angle (DCA). The thickness is measured using digital callipers, and the resistance to moisture transfer is measured using a Permatran-w 101K (Ametek Mocon), a device for measuring water vapour transmission rate through a film. The thermal conductivity of the membrane is given by the manufacturer. The thickness, thermal conductivity and the resistance to moisture transfer are used in the analytical model to calculate the heat and moisture transfer rates through the membrane. The DCA represents the wettability of the surface, which indicates if the membrane is hydrophobic or hydrophilic. An angle of less than 90° corresponds to a hydrophilic surface, and an angle greater than 90° corresponds to a hydrophobic surface. The DCA is measured using a drop shape analyser (KRŰSS Scientific). The Propore^TM^ membrane is slightly hydrophobic, with a DCA of 92°.

**Table 1.** Specifications of the Propore^TM^ membrane [4,7].

property	value
thickness (*L*)	0.20 mm
thermal conductivity (*k*_mem_)	0.34 W m^-1^ K^-1^)
resistance to moisture (*R_m,_*_mem_)	140 s m^−1^
DCA	92°

**Figure 2.**
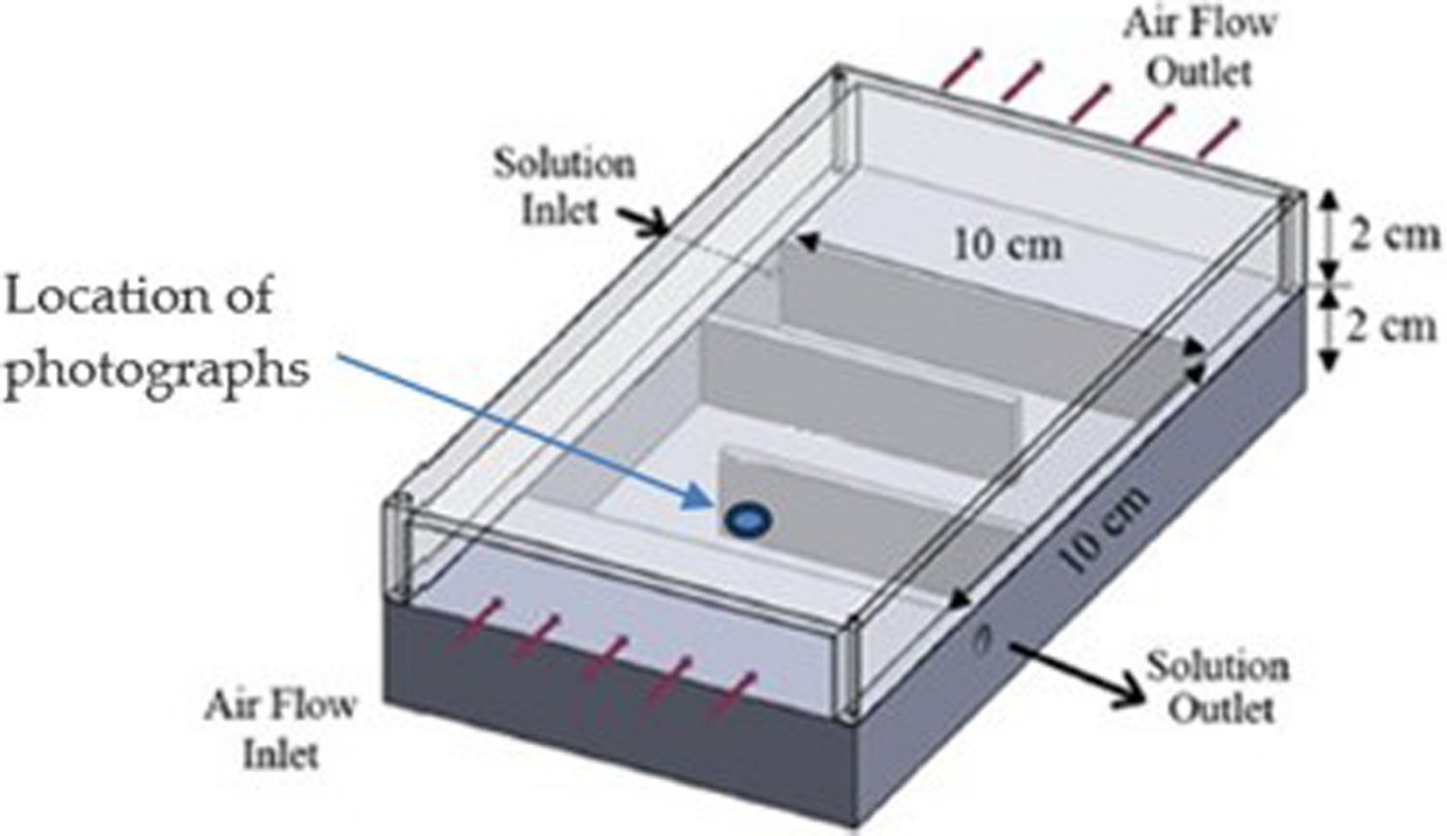
Schematic of test section used for frost growth tests. The LD circulates through the bottom portion and the air flow crosses over the top portion.

The tests are conducted on a small-scale counter-crossflow LAMEE, as shown in figure 2. The bottom tray holds the LD and air flows through the channel on top. The membrane is not shown (to make clear the liquid channels) but would be attached to the surface halfway up the height of the device, on the top of the baffles. The baffles create channels for the LD to flow through, avoiding stagnation areas in the corners of the tray, as well as providing additional surfaces on which to glue the membrane.

The air flow and LD are pre-conditioned and enter the test facility at a set air temperature, RH and flow rate, as well as LD temperature, concentration and flow rate. The temperatures are measured using Type-T thermocouples, calibrated using a dry-well calibrator (Hart Scientific 9107) and have an uncertainty of ± 0.2°C. The air RH is measured using HIH-4010 humidity sensors (Honeywell) calibrated using a Mini Humidity Generator (Thunder Scientific, Model 1200) which has an uncertainty of ± 1% RH for the experiments conducted with room temperature air. For the experiments conducted with air at 0°C, the RH sensors were calibrated using the humidity fixed point method [8] and have a higher uncertainty of ± 5% RH. The uncertainty of the sensors is expected to be higher at low air temperatures. The density of the LD is measured using a density meter (Anton Paar, DMA 4500M) and converted to a concentration. The air temperature and RH are measured upstream and downstream of the test section, to ensure good mixing to allow the bulk values to be measured. The temperature of the LD is measured with thermocouples inserted through the base of the test section. The density of the LD is measured by withdrawing a sample from the tray before and after each test; it is not measured throughout the test. Photographs of frost are taken at the inlet of the air stream, just as it comes in contact with the membrane, which is the leading edge of the energy exchanger, as shown in figure 2.

The test section has dimensions of 10 × 10 cm, with the air channel and the LD channel each having a height of 2 cm. The hydraulic diameter of the test section is 0.03 m. The temperature and RH of the air are measured at both the inlet and outlet, in multiple locations across the channel, and the average values at the inlet and outlet are calculated. The LD temperature is measured at two locations, and the reported value is the average of the two readings. The concentration of the LD is measured by withdrawing samples and testing them in the density meter. The equilibrium air humidity ratio of the LD can be calculated from the known temperature and concentration [9]. The air side of the test section is made of clear acrylic to allow photographs of the membrane and the condensation/frost that accumulates on the surface.

### (b) Analytical model

**Figure 3.**
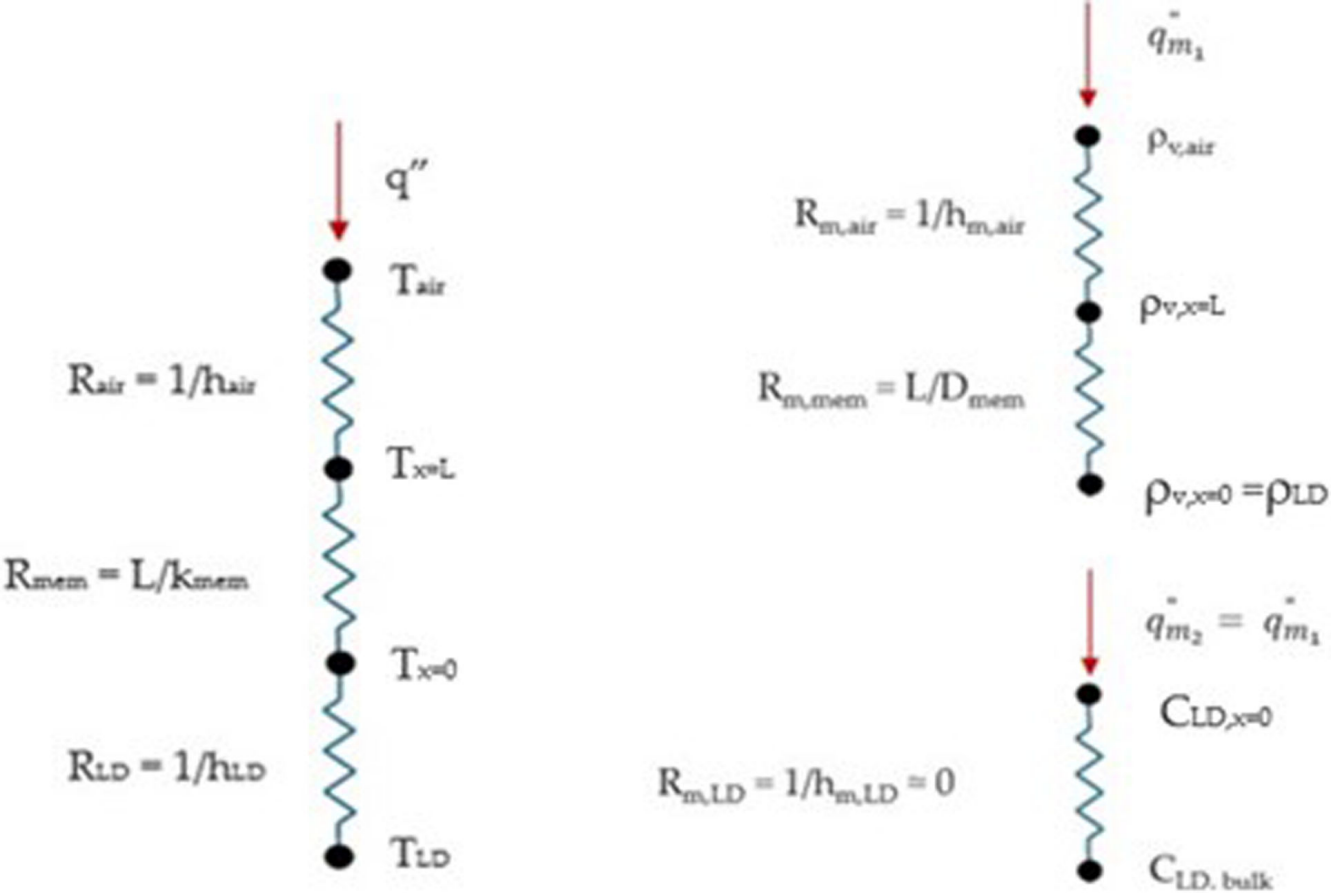
Thermal and moisture resistance networks for a membrane separating air and a LD.

An analytical model to determine the onset of frost on the surface of a porous membrane has been developed [5] based on one-dimensional heat and moisture transfer through the membrane, as shown by the resistance networks presented in figure 3. It is assumed that the temperature and humidity profiles inside the membrane are linear at steady-state conditions, allowing an analytical model to be used [6]. The purpose of the analytical model is to determine the LD temperature that will result in a RH of 100% RH or greater on the top surface of the membrane, for a given air temperature, RH and LD concentration. If the temperature of the top surface of the membrane is below 0°C, this condition will result in frost growth on the membrane surface.

The heat flux through the membrane (*q″*) is a function of temperature (*T*) at different locations in the network and the resistance to heat transfer of the air (*R*_air_), membrane (*R*_mem_) and LD (*R*_LD_), as given in equation (2.1).


(2.1) $$q^{\prime \prime }=\frac{T_{\text{air}}-T_{x=L}}{R_{\text{air}}}=\frac{T_{x=L}-T_{x=0}}{R_{\text{mem}}}=\frac{T_{x=0}-T_{LD}}{R_{\text{LD}}}.$$

The subscripts in equation (2.1) refer to the bottom surface of the membrane (*x = 0*), in contact with the LD and the top surface of the membrane (*x = L*), in contact with the air, as indicated in figure 3. The moisture transfer through the membrane (${\overset{˙}{m}}_{v}^{\prime \prime }$) is a function of the vapour density (*ρ_v_*) at different locations in the network and the resistance to moisture transfer of the air (*R_m_*_,air_) and membrane (*R_m_*_,mem_), given in equation (2.2). The moisture transfer resistance of the LD is assumed to be negligible [10]:


(2.2) $${\overset{˙}{m}}_{v}^{\prime \prime }=\frac{\rho_{v,\text{air}}-\rho_{v,x=L}}{R_{m,\text{air}}}=\frac{\rho_{v,x=L}-\rho_{v,x=0}}{R_{m,\text{mem}}}.$$

To convert between RH and vapour density of air, equations (2.3) and (2.4) are used


(2.3) $$\rho_{v}=\frac{p_{v}}{R_{v}T},$$

(2.4) $$\text{RH}=\frac{p_{v}}{p_{\text{sat}}},$$

where *p_v_* is the vapour pressure, *R_v_* is the specific gas constant and *p*_sat_ is the saturation pressure, which is a function of temperature. Equations (2.1) and (2.2) are reorganized to solve for the conditions at the top surface, *T_x = L_* and *ρ_v,x = L_*, as shown in equations (2.5) and (2.6)


(2.5) $$T_{x=L}=\frac{\left(R_{\text{mem}}+R_{\text{LD}}\right)T_{\text{air}}+R_{\text{air}}T_{\text{LD}}}{R_{\text{air}}+R_{\text{mem}}+R_{\text{LD}}},$$

(2.6) $$\rho_{v,x=L}=\frac{R_{m,\text{mem}}\rho_{v,\text{air}}+R_{m,\text{air}}\rho_{v,x=0}}{R_{m,\text{air}}+R_{m,\text{mem}}}.$$

The heat-transfer resistance of the membrane (*R*_mem_) is calculated from the thickness (*L*) and thermal conductivity (*k*_mem_) of the membrane. The resistance of the membrane to moisture transfer is measured directly. The heat-transfer coefficients (*h*) in the air and LD are calculated using correlations found in the literature for the specific geometry and air-flow conditions of the test section. In a LAMEE, the membrane may be oriented in either a horizontal or a vertical direction. If the membrane is in the horizontal direction, forced convection will generally be dominant, and correlations for thermally developing laminar flow through a rectangular duct under forced convection, with a constant temperature on one wall, can be used to find the local Nusselt number and the local heat-transfer coefficient [11,12]. In the case of forced convection, Nusselt number (Nu) will be a function of Reynolds number (Re) and Prandtl number (Pr), [Nu *= f* (Re*,* Pr)].

If the membrane is positioned in the vertical direction, natural convection may be significant and must be considered. In this case, Nusselt number correlations for natural convection must be used [13] and combined with the forced convection Nusselt number to give a mixed convection Nusselt number. For natural convection, the Nusselt number will be a function of Rayleigh number (Ra), which is the product of Grashof number (Gr) and Prandtl number (Pr), [Nu *= f (*Ra*) = f (*Gr*,* Pr)].

The heat-transfer coefficient has been found to have a significant effect on the results of the analytical model, so care must be taken when determining the correct correlations for the Nusselt number. Due to the thermally developing boundary layer, the Nusselt number and heat-transfer coefficient will vary across the length of the exchanger, being highest at the inlet and decreasing towards the outlet. Correlations for the local Nusselt number and local heat-transfer coefficients should be used, and the heat-transfer coefficient can be calculated for a location just after the entrance (e.g. *x* = 0.01 cm). Alternatively, the average Nusselt number and heat-transfer coefficient over the observation region can be used, both of these approaches are reasonably accurate. When the calculated heat-transfer coefficient is used as the input for the model, the model will predict whether frost occurs at that specific location on the membrane surface.

The convective moisture transfer coefficient of the air side (*h_m_*_,air_) is calculated from heat- and mass-transfer analogies using the air-side heat-transfer coefficient. The convective moisture transfer resistance on the LD side is assumed to be zero [10]. The vapour density of the air (*ρ_v_*_,air_) is calculated from the temperature and RH of the air, assuming ideal gas behaviour for the air. At the bottom surface of the membrane, the vapour density of the air must be equal to the equilibrium vapour density of the LD, which is a function of the temperature (*T*_LD_) and concentration of the LD (*C*_LD_) [14], which are measured in the experiments. The equilibrium vapour density of the LD can be presented as an equilibrium air humidity ratio for the LD, similar to the humidity ratio of the air.

### (c) Frost limits

The conditions at which frost occurs in the membrane can be calculated using the model, to create maps of the frost limit of the LAMEE. The properties of the membrane and the air and LD conditions are used for input to the model. Using the resistance networks shown, the temperature and RH of the top surface of the membrane are calculated. If the RH is equal to 100% RH and the temperature is less than or equal to 0°, it is assumed that frost will occur. The model can be used to create maps of when frost will occur, by varying the air temperature and RH and the LD temperature. Each frost map is specific to the membrane tested, as the results will be affected by the moisture transfer resistance of the membrane. If a membrane with a lower resistance to moisture transfer is used, more moisture transfer will occur through the membrane, reducing the likelihood of frost under the same conditions.

## 3. Results and discussion

### (a) Frost growth on a porous membrane

To study how frost grows on a porous membrane, photographs of the membrane surface were taken at different time steps, using a digital camera. The test was also conducted on a hydrophobic (DCA = 106°) impermeable plastic sheet in place of the membrane, to determine the effect that the moisture transfer through the membrane has on reducing frost growth. For this test, the air temperature was set to 22.0°C, air humidity ratio to 7.0 g_w_/kg_air_ (43% RH), LD temperature to −7.5°C and an equilibrium air humidity ratio of the LD of 1.3 g_w_/kg_air_. Photographs were manually taken every 30 s and analysed after the whole test was complete. Image analysis software was used to help detect the presence of condensation and/or frost by comparing images of the bare membrane with the images taken during the test.

For the impermeable plate, condensation droplets formed almost immediately after the test began, and the droplets began to freeze in less than 5 min. Within 15 min, the whole surface of the impermeable plate was covered with frost. For the porous membrane, the first signs of condensation did not occur until after 15 min, and the first signs of frost occurred after 40 min. Even though the two surfaces were at the same temperature and below 0°C, frost did not form on the porous membrane as quickly as on the impermeable plate due to the moisture transfer through the membrane. The moisture transfer through the membrane reduces the vapour pressure of the air directly above the surface of the membrane, lower than it would be for an impermeable surface. As such, the rate of frost nucleation is slower on a membrane than on an impermeable surface [15]. This means that the time needed for frost to grow to an observable size is longer on a membrane, and in an energy exchanger, it will take more time before the frost grows enough to affect the performance of the exchanger.

**Figure 4.**
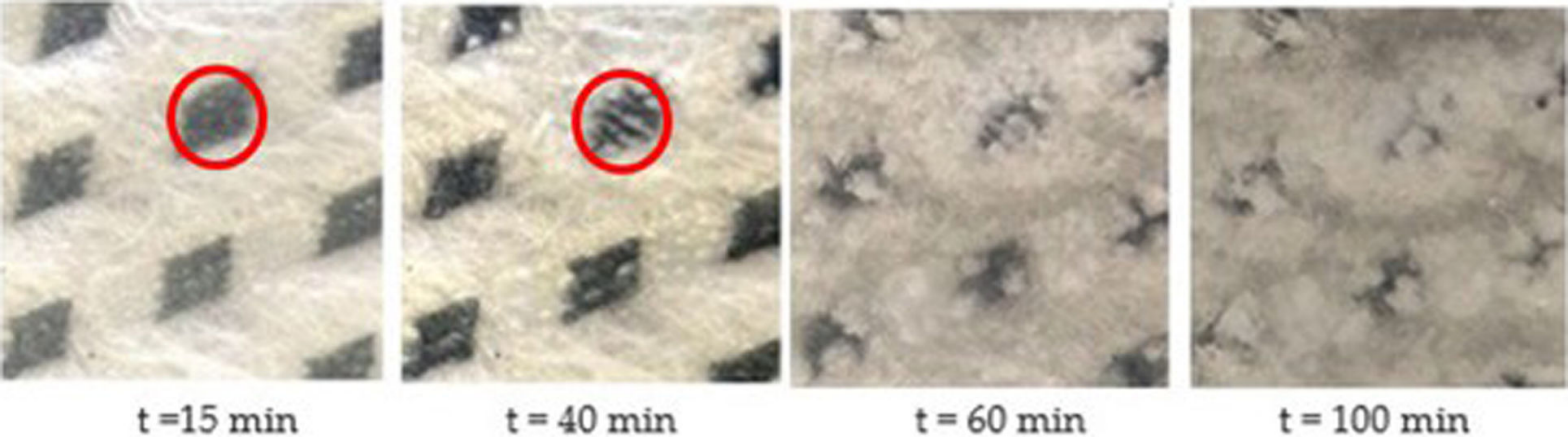
Photographs of the leading edge of the porous membrane after *t* = 15, 40, 60 and 120 min, with *T*_air_ = 22.0°C, *W*_air_ = 7.0 g_w_/kg_air_ (43% RH), *T*_LD_ = −7.5°C and *W*_LD_ = 1.3 g_w_/kg_air_.

Photographs of the frost growth on a porous membrane in the transient test described are shown in figure 4. Each picture is taken at the same location, at the leading edge of the membrane, when the air flow first meets the cold membrane surface. The first photograph shows the membrane surface 15 min after the start of the test, and small droplets can be seen on the surface. The second photograph shows the membrane surface after 40 min, where the condensation has turned into frost crystals. As the test continues, the frost spreads to other parts of the membrane and begins to grow thicker and more dense. These photographs show the leading edge of the membrane; farther downstream, there is less frost, and, in some conditions, no frost at all.

### (b) Frost limits

**Figure 5.**
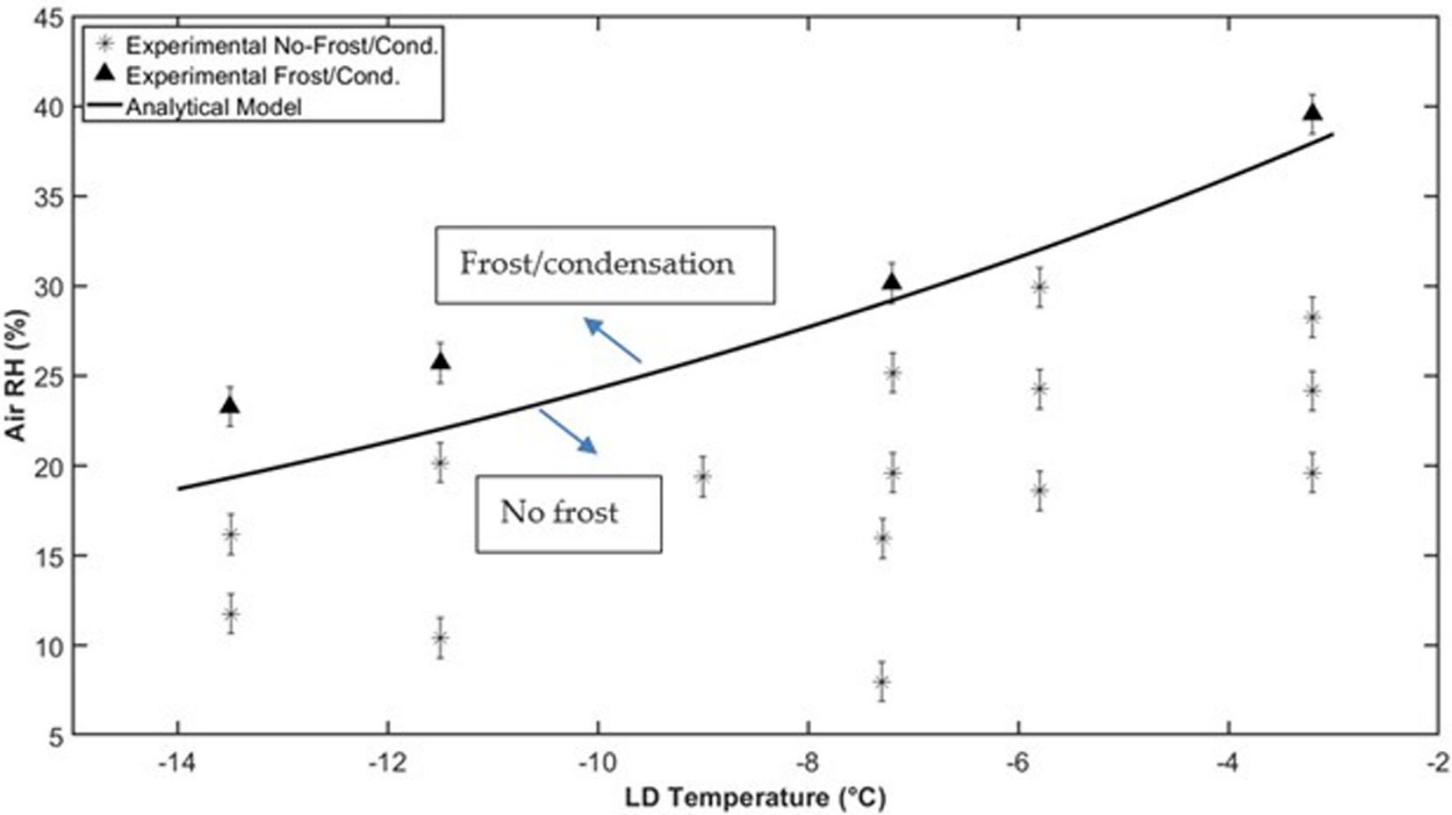
Experimental and numerical frost limits for a Propore^TM^ membrane at *T*_air_ = 23°C and Re_air_ = 190.

**Figure 6.**
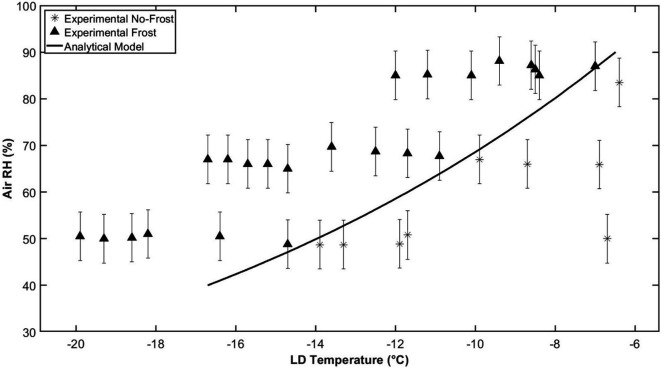
Experimental and numerical frost limits for a Propore^TM^ membrane at *T*_air_ = 0°C, with Re_air_ = 177, Ra_air_ = 2.6 × 10^5^, Re_LD_ = 4 and Ra_LD_ = 1.7 × 10^6^.

It has been shown that the moisture transfer through the membrane leads to a delay in the time it takes for frost to form on the membrane surface. There is also a shift in the conditions at which frost will occur on the surface, which is referred to as the frost limit. The frost limit is defined as the lowest air humidity ratio at a constant LD temperature or the highest LD temperature at a constant air humidity ratio, which leads to frost growth on the membrane. The frost limit can be determined both experimentally and through the analytical model. Figures 5 and 6 show the frost limits for a Propore^TM^ membrane with varying LD temperatures and air RHs for both the experiments and the analytical model. Two of the significant variables that affect the frost limits, in addition to the resistance to moisture transfer (*R_m_*_,mem_), are the air temperature and the heat-transfer coefficients of the air and LD. The experiment shown in figure 5 was conducted at an air temperature of 23°C, with forced convection being dominant. The experiment shown in figure 6 was conducted at an air temperature of 0°C, with mixed convection conditions. Figure 5 is adapted from [5], where the experimental data were used to validate the analytical model. In figure 6, new experimental data are presented and show that the model is also reasonably accurate at air temperatures down to at least 0°C.

Two different strategies were employed in these experiments to obtain the frost limit under the given operating conditions. In the tests with an air temperature of 23°C (figure 5), the strategy was to hold the LD temperature constant and gradually increase the air RH, until frost appeared on the membrane. For the tests at 0°C (figure 6), the strategy was to hold the RH of the air constant and reduce the LD temperature until the first signs of condensation and then frost appeared. Both strategies are valid and result in the same frost limits. The aim of the experiments is to hold one variable (air RH or LD temperature) constant and change the other variable in small increments to accurately determine the point where frost first forms. The choice of which variable to change in small increments depends on the test facility and instrumentation and which variable can be accurately changed in small increments. It was found that holding the RH constant and changing LD temperature very gradually was easier to do, but both will give the same result for the frost limit map and both have good agreement with the analytical model.

Figure 5 shows the frost limit for an air temperature of 23°C, with forced convection conditions (Re = 190). This map shows the conditions (LD temperature and air RH) that will lead to frost on the surface of the porous membrane. The points marked by asterisks did not result in frost on the surface, while the points marked by triangles did result in frost on the surface. The solid line shows the results from the analytical model. Any points below and to the right of the line do not result in frost on the surface. Points above and to the left of the line do result in condensation or frost on the surface. There is good agreement between the model and the experiments at LD temperatures of approximately −3.5°C and −7°C. It was found during testing that it was difficult to control the air RH to very fine increments, so it was difficult to get to the point where frost first appeared, which is represented by the model. However, all points where frost occurred in the experiments are above the analytical model line, and all points where frost did not occur are below the line. The uncertainty in the air RH is approximately ± 1.1% RH, as shown by the error bars on the graph. The uncertainty in the LD temperature is approximately ±0.2°C, which is too small to be seen on the graph. Compared to experiments under the same conditions (air at 23°C and 12% RH) on an impermeable surface, it was found that the LD temperature could be 2°C–3°C lower when the membrane was used before signs of frost were detected, confirming that the moisture transfer through the porous membrane can help to reduce the amount of frost that will form on the surface [5]. These frost limit maps can be used to help designers find acceptable operating conditions for energy exchangers to avoid frost formation inside the exchanger.

Figure 6 shows the frost limit for an air temperature of 0°C, with mixed convection conditions, where the test section is placed in the vertical position and natural convection is significant. In this case, the flow conditions in both the air side and the liquid side are important, because of the natural convection. The air side has Re = 177 and Ra = 2.6 × 10^5^, and the LD side has Re = 4 and Ra = 1.7 × 10^6^; Ra varies with the temperature of the LD; the numbers presented are for the median LD temperature of −15°C. As before, the points where no frost occurred are shown by asterisks, and the points where frost is present on the surface are shown by triangles. In this test, the LD temperature was gradually reduced until signs of frost were seen on the surface of the membrane. It was found that it was easier to control the LD temperature in gradual increments, so there is better agreement between the experimental results and the analytical model, shown by the solid line. The uncertainty in the LD temperature is still ± 0.2°C; however, the uncertainty in the air RH is ± 5.2% RH, as the uncertainty gets higher at lower air temperatures.

The frost limit maps presented can be used by designers to develop frost-free energy exchangers, by designing the exchangers to work in the ‘no frost’ zone represented on the map. The use of a membrane-based energy exchanger allows the energy exchanger to operate in a wider range of conditions without frost, due to the moisture transfer through the membrane and the reduction in vapour pressure at the surface of the membrane, which results in a slower rate of frost growth on the surface. The analytical model can be used by designers to create these frost limits for various operating conditions, without the need for expensive experiments. However, care must be taken to accurately quantify the heat and moisture transfer coefficients in the air and LD streams, as well as the moisture transfer resistance of the membrane, to get accurate results from the model. The analytical model was previously presented and validated under forced convection conditions, and in this paper is also validated under a combination of forced and natural convection conditions. With careful use of this analytical model, designers will be able to get closer to the design of a frost-free energy exchanger.

## 4. Conclusions

LAMEEs are devices that can transfer both heat and moisture between an air stream and a liquid stream and have important applications in humidification and dehumidification of air. Experimental tests have been conducted and an analytical model developed to study the effects of heat and moisture transfer through membranes on frost growth in cold conditions. This paper presents a review of frost growth on membranes for air temperatures at or near room temperature (23°C) and extends the findings with new research on air temperatures as low as 0°C. Experimental frost limit maps have been developed for these air temperatures and used to verify the analytical model, which can be used to predict the frost limits under other conditions. The frost limits are dependent on several variables, including air temperature, and the heat and moisture transfer coefficients in the air and LD. To obtain good agreement between the analytical model and the experimental results, it is important to accurately quantify these coefficients. In conclusion, the use of a porous membrane in an energy exchanger can have a significant effect on the formation of frost on the surface of an exchanger, which is very beneficial to exchanger performance. The development of frost-free energy exchangers would make a significant effect on the energy exchanger industry, and membrane-based energy exchangers are a step towards this goal.

## Data Availability

A link to the dataset is provided here: [16].
